# Temporal variations in maternal treatment requirements and early neonatal outcomes in patients with gestational diabetes

**DOI:** 10.1111/dme.14596

**Published:** 2021-05-19

**Authors:** Rachel A. Fox, Charlotte J. Patient, Abigail R. Aiken, Claire L. Meek, Catherine E. Aiken

**Affiliations:** 1 School of Clinical Medicine, Addenbrooke’s Hospital, Cambridge, UK; 2 Department of Obstetrics and Gynaecology, Rosie Hospital, Cambridge University Hospitals, Cambridge, UK; 3 LBJ School of Public Affairs, University of Texas at Austin, Austin, TX, USA; 4 Institute of Metabolic Science, Addenbrooke’s Hospital, Cambridge, UK; 5 Department of Clinical Biochemistry, Cambridge University Hospitals, Addenbrooke’s Hospital, Cambridge, UK; 6 Wolfson Diabetes and Endocrinology Clinic, Cambridge University Hospitals, Addenbrooke’s Hospital, Cambridge, UK; 7 University Department of Obstetrics and Gynaecology, University of Cambridge, NIHR Cambridge Comprehensive Biomedical Research Centre, Cambridge, UK

**Keywords:** gestational diabetes, pregnancy, temporality, seasonality, birthweight, obstetric outcomes, climate

## Abstract

**Aims:**

There is seasonal variation in the incidence of gestational diabetes (GDM) and delivery outcomes of affected patients. We assessed whether there was also evidence of temporal variation in maternal treatment requirements and early neonatal outcomes.

**Methods:**

We performed a retrospective analysis of women diagnosed with GDM (75 g oral glucose tolerance test, 0 h ≥ 5.1; 1 h ≥ 10.0; 2 h ≥ 8.5 mmol/L) in a UK tertiary obstetric centre (2015–2019) with a singleton infant. Data regarding demographic characteristics, total insulin requirements and neonatal outcomes were extracted from contemporaneous electronic medical records. Linear/logistic regression models using month of the year as a predictor of outcomes were used to assess annual variation.

**Results:**

In all, 791 women (50.6% receiving pharmacological treatment) and 790 neonates were included. The likelihood of requiring insulin treatment was highest in November (*p* < 0.05). The average total daily insulin dose was higher at peak (January) compared to average by 19 units/day (*p* < 0.05). There was no temporal variation in neonatal intensive care admission, or neonatal capillary blood glucose. However, rates of neonatal hypoglycaemia (defined as <2.6 mmol/L) were highest in December (40% above average; *p* < 0.05).

**Conclusions:**

Women with GDM diagnosed in winter are more likely to require insulin treatment and to require higher insulin doses. Neonates born to winter-diagnosed mothers had a corresponding increased risk of neonatal hypoglycaemia. Maternal treatment requirements and neonatal outcomes of GDM vary significantly throughout the year, even in a relatively temperate climate.

## 1 | INTRODUCTION

Evidence suggests that the likelihood of developing gestational diabetes (GDM) is influenced by external environmental factors, including variation by season. Seasonal differences in GDM risk have been demonstrated in diverse climates around the world from temperate to subtropical, including North America,^1^ South America,^2^ Australia,^3^ Asia^4^ and Europe.^5,6^ Globally, there is a consistently higher incidence of GDM in hotter months. The reason for temporal variation in GDM incidence is not fully understood but could reflect either the impact of ambient temperature on blood glucose measurement^7^ or seasonal differences in diet and lifestyle.

Recent evidence from our centre suggests that pregnancy outcomes also vary seasonally in pregnancies affected by GDM,^5^ with higher birthweights and a corresponding increase in the risk of emergency Caesarean section during colder months. However, it is not known whether maternal treatment requirements or neonatal outcomes in GDM vary throughout the year.

Evidence suggests that insulin resistance at GDM diagnosis is highest in colder months.^3^ Higher insulin resistance at diagnosis is associated with increased requirement for insulin and higher mean maximum daily insulin dose,^8^ suggesting that GDM treatment requirements might be greater in colder months. However, post-load glucose levels (assessed using oral glucose tolerance test; OGTT) tend to be higher in warmer months.^2,3,6^ As increased post-load glucose levels are also associated with increased insulin requirements in GDM,^9^ it could also be the case that the need for insulin treatment may be reduced in colder months.

As proactive intervention to control maternal hyperglycaemia leads to improved outcomes,^10^ an understanding of differing treatment requirements by season could be valuable in personalising care for women with GDM. We aimed to assess whether there is temporal variation in the treatment requirements and neonatal outcomes of women diagnosed with GDM in the context of a relatively temperate climate in the United Kingdom.

## 2 | METHODS

A cohort of 791 women diagnosed with GDM who delivered singleton infants at term (37–42 weeks of gestation) from January 2015 to January 2019 at The Rosie hospital, Cambridge was identified as part of a service evaluation which had prospective institutional approval. The records of 790 neonates were also available for analysis. Detailed hand-searching of the mothers’ and babies’ electronic medical records enabled collection of data regarding treatment and maternal and neonatal outcomes. The data were extracted retrospectively from routine documentation of patient encounters and were not collected specifically for study purposes. Where a woman had two or more full-term pregnancies affected by Gestational diabetes (GDM) and otherwise eligible for inclusion within the study window, we only included the first pregnancy in the analysis. The primary outcome of this study was maternal treatment modality for GDM, specifically treatment with insulin. We included other details of the treatment regimen (total units of insulin), neonatal hypoglycaemia and neonatal lowest blood glucose as secondary outcomes.

Diagnosis of GDM was made based upon International Association of Diabetes in Pregnancy Study Groups (IADPSG) recommendations (75 g OGTT, 0 h ≥ 5.1; 1 h ≥ 10.0; 2 h ≥ 8.5 mmol/L).^11^ Women with risk factors for GDM were offered a 75 g OGTT at 24–28 weeks as per NICE guidelines,^12^ and additional OGTTs were offered to women on an ad hoc basis, for example based on results of random plasma glucose tests or growth scans. Women with previous GDM were offered capillary glucose self-monitoring or 75 g OGTT as soon as possible after booking (usually 11–16 weeks of gestation).

After diagnosis of GDM, women were advised to take regular capillary glucose measurements and given detailed dietary and lifestyle advice. Women with persistent hyperglycaemia despite dietary and lifestyle changes were offered escalating treatment, using metformin and/or insulin, to achieve target capillary glucose of fasting level <5.3 mmol/L and 1-hour postprandial level <7.8 mmol/L.

After delivery, all infants had pre-feed capillary glucose readings taken from 4 h after birth until there were three consecutive pre-feed readings ≥2.6 mmol/L. Readings <2.6 mmol/L were considered consistent with neonatal hypoglycaemia and treated with increased breastfeeding, expressed breast milk or formula until target blood glucose levels were met or treatment escalated, for example with IV dextrose.

Maternal demographic data collected included maternal age, ethnicity, BMI at booking and smoking status during pregnancy. Obstetric history included previous GDM, parity (collapsed into 0, 1 or ≥2) and previous delivery mode (caesarean section or vaginal delivery). Gestation at diagnosis and results of OGTT and HbA1c at diagnosis were recorded. Treatment data consisted of treatment modality (diet, met-formin, insulin or both) and maximal dosage required (for pragmatic reasons taken to be at 36 weeks). 14-day average blood glucose readings (mmol/L) and gestational weight gain (kg) were recorded for all women at the time of each appointment. Delivery variables were delivery mode (spontaneous or instrumental vaginal delivery, or caesarean section) and estimated blood loss (ml).

Neonatal outcomes included sex, birthweight, neonatal capillary glucose readings, neonatal hypoglycaemia, jaundice and admission to neonatal intensive care unit (NICU). Birthweight centiles were adjusted for gestational age and sex, using Intergrowth 21st standards.^13^

Group-wise comparisons were carried out using either Student’s *t*-test or the Mann–Whitney test for numerical data, and Pearson’s chi-squared test for categorical data. We analysed the likelihood of each outcome of interest dependent on the month of the year. We ran two separate models for maternal and neonatal outcomes. For maternal treatment outcomes, the day of interest was at 28 weeks. This day was selected to best reflect the date that GDM would have been diagnosed and maternal treatment decisions would have been made for all patients in the cohort (accepting that for some women, treatment may be altered up to and beyond 36 weeks). For neonatal outcomes, the day of interest was the day of delivery. This day was selected to best represent the day on which the neonatal outcome occurred, accepting that for some babies there may be a delay between delivery and outcome. Linear or logistic regression models were constructed for each outcome of interest with a dummy variable for month of the year. All models were adjusted for individual covariates, which were selected to optimise the Akaike information criterion; booking BMI, ethnicity and parity.

As a secondary analysis, generalised additive modelling (GAM) was used to allow non-parametric model fitting with relaxed assumptions about the functional form of the relationship between day of the year, treated as a continuous function, and outcomes of interest. The models were constructed by iteratively fitting weighted additive models through backfitting and were specified using the R package ‘gam’ (https://cran.r-project.org/web/packages/gam/gam.pdf ). The backfitting algorithm is a Gauss–Seidel method for fitting additive models by iteratively smoothing partial residuals. Our models incorporated a nonlinear term for the effect of day of year on the risk of each outcome, estimated using cubic splines, and were adjusted for booking BMI, ethnicity and parity. The risk of any given outcome for each day of the year is compared to the average risk calculated over the entire study period, and visually represented on the figure plots. Caution should be exercised regarding the interpretation of the GAM model estimates for the first and last few days of the year, as the modelling estimates rely on a neighbourhood of data from either side of the point of interest. This is absent for one side of data at the extremes of the *x*-axis, increasing the uncertainty around these estimates and increasing the potential for bias. As a sensitivity analysis, we also ran models with day 1 designated as the 1st July rather than the 1st January. This did not substantively alter the results of our analyses (Figure S1).

Statistical significance of the nonlinear effect of day of year was assessed using a likelihood-ratio test.

Findings were considered statistically significant at an alpha level of 0.05. All analyses were conducted using the R statistical software package version 3.5.1.^14^ A ‘complete cases’ approach was adopted to missing data. Analysis based on BMI recordings later in pregnancy suggested that booking BMI values were likely to be missing at completely random in the study population.

## 3 | RESULTS

### 3.1 | Participant characteristics

In all, 400/791 (50.6%) of women required pharmacological treatment for their GDM at 36 weeks. 11.8% were treated with metformin alone, 24.7% with insulin alone and 14.2% with both insulin and metformin (Table 1). The average birthweight in the cohort was 3308 g (±425 g; Table 2).

### 3.2 | Treatment of GDM

**FIGURE 1:**
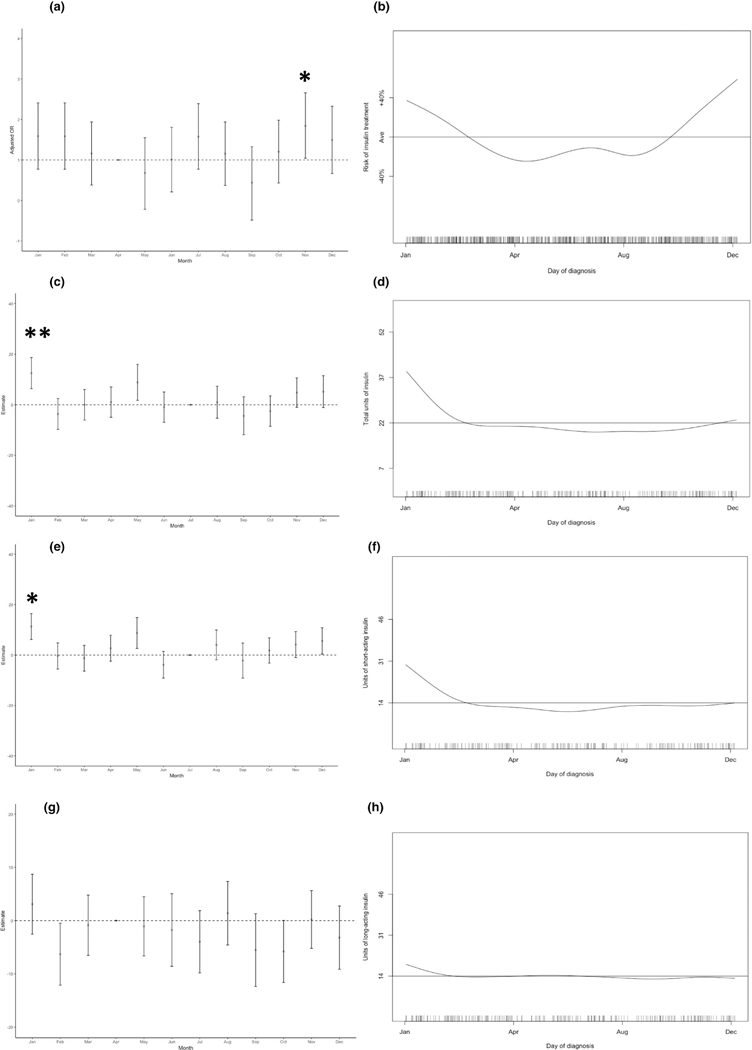
The impact of temporal variation upon requirement for insulin treatment (a, by month; b, using generalised additive modelling [GAM], *p*[overall trend] <0.05), total number of units of insulin required (c, by month; d, GAM, *p*[overall trend] <0.05), number of units of short-acting insulin required (e, by month; f, GAM, *p*[overall trend] =0.07), number of units of long-acting insulin required (g, by month; h, GAM, *p*[overall trend] >0.1), All analyses were adjusted for booking BMI, ethnicity and parity. Horizontal lines represent the average taken across the entire year. Tick marks on the *x*-axis of GAM figures represent the number of individual data points included on each day of the year

The average glucose readings in the first 2 weeks post-diagnosis did not vary temporally and there was no temporal difference in gestational weight gain. The likelihood of requiring treatment with diet or metformin did not vary temporally. However, the likelihood of requiring treatment with insulin did vary, being significantly increased for women who were 28 weeks of gestation in November (*p* < 0.05; Figure 1a). The likelihood of requiring insulin treatment was >40% above baseline November–January and >10% below baseline March–August (*p* < 0.05 for overall trend; Figure 1b).

The total insulin requirement among women treated with insulin (*n* = 307) was significantly increased in women who were 28 weeks of gestation in January (*p* < 0.01; Figure 1c). The total insulin requirement in the cohort varied by 19 units/day from the highest requirement in January to the lowest in July (*p* < 0.05 for overall trend; Figure 1d). We also assessed the effects upon short-acting and long-acting insulin use. The number of units of short-acting insulin given per day was significantly higher in women who were 28 weeks of gestation in January (*p* < 0.05; Figure 1e). When assessed by over-all trend throughout the year, short-acting insulin requirement varied by 16 units from greatest (January) to lowest (July) but this variation was not statistically significant (*p* = 0.07, *n* = 171; Figure 1f). There was no significant temporal variation in the number of units of long-acting insulin given per day (*n* = 268; Figure 1g,h).

### 3.3 | Obstetric and neonatal outcomes

There was no annual variation in birthweight nor in the number of large for gestational age babies (birthweight greater than 90th centile). The risk of delivery by emergency caesarean section did not vary temporally.

**FIGURE 2:**
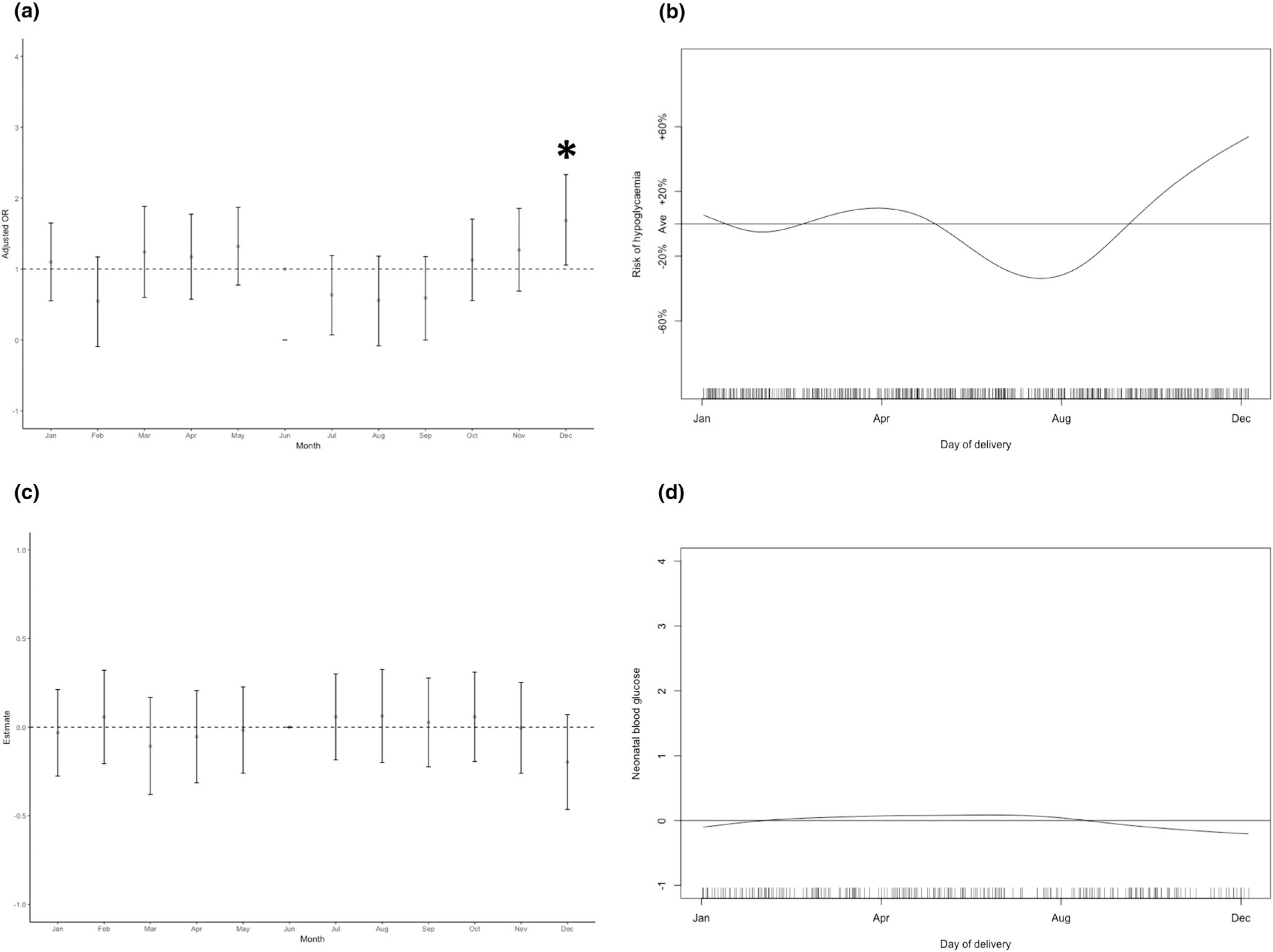
Temporal variation in risk of neonatal hypoglycaemia (a, by month; b, GAM, *p*[overall trend] <0.05), and lowest measured neonatal blood glucose concentration (c, by month; d, GAM, *p*[overall trend] >0.1). All analyses were adjusted for booking BMI, ethnicity and parity. Horizontal lines represent the average taken across the entire year. Tick marks on the *x*-axis of GAM figures represent the number of individual data points included on each day of the year

The risk of neonatal hypoglycaemia was significantly increased in babies delivered in December (*p* < 0.05; Figure 2a). When analysed as a continuous variable, the risk of hypoglycaemia varied by day of delivery from a high of 40% above baseline in December to a low of 30% below baseline in July (*p* < 0.05, Figure 2b). There was no annual variation in the number of babies admitted to NICU for any cause. When analysed either by month or as a continuous variable, neonatal capillary glucose did not show temporal variation (±0.15 mmol/L; Figure 2c,d).

## 4 | DISCUSSION

We show that women diagnosed with GDM in winter months are more likely to require treatment with insulin and to need 19 units/day more insulin, compared to women diagnosed in summer months. Women who delivered their baby in the summer months had the lowest GDM treatment requirements and a correspondingly lower risk of having a neonate with hypoglycaemia.

### 4.1 | Maternal and neonatal outcomes

The lack of temporal variation of maternal and neonatal outcomes we found (with the exception of neonatal hypoglycaemia) is reassuring as it suggests that escalating treatments at high-risk times may help to achieve the same outcomes throughout the year. Treatment for GDM is adjusted throughout pregnancy to achieve optimal glycaemic control. Addition of insulin occurs only when lifestyle modifications are not sufficient to keep blood glucose within the target range, which could result from greater insulin resistance or reduced engagement with non-pharmacological treatments such as diet and exercise. Increased risk of treatment with insulin may also be due to the effect of temperature on OGTT results causing differences in average severity of GDM diagnosed in winter compared to summer. A previous study at our centre reported significant seasonal variation in birthweight and rates of emergency caesarean section in GDM pregnancies.^5^ The period of data collection was earlier (2004–2008) than ours (2015–2019), and the diagnostic criteria used were WHO 1999 and modified WHO 1999^15,16^ rather than IADPSG criteria used in our study. It is possible that the temporal variations in pharmacological management observed in the current cohort have eliminated the previous variations in clinical outcomes, but it is not possible to test this directly, due to lack of detailed information about treatment dosages in 2004–2008.

Temporal variation in maternal hyperglycaemia and hence treatment requirement could impact risk of neonatal hypoglycaemia. There are conflicting reports about whether maternal treatment affects the risk of neonatal hypoglycaemia with some studies finding increased risk if mothers required medical treatment^17^ while others find no significant difference in risk between neonates of diet-treated and insulin-treated mothers.^18^ Although we found significant temporal variation in the risk of neonatal hypoglycaemia when treated as a categorical variable, it is important to note the annual range of mean lowest capillary glucose reading was <0.15 mmol/L and there was no significant temporal variation. Since the risk of adverse neurodevelopmental outcomes of hypoglycaemia depends upon severity of hypoglycaemia,^19^ it is doubtful whether such a small absolute difference would translate to a meaningful temporal difference in the most severe consequences of neonatal hypoglycaemia, especially given that there was no temporal difference in rate of admission to NICU.

### 4.2 | Insulin resistance

Seasonal variation in glycaemic control has been demonstrated in school-aged children with type 1 diabetes ^20^ and adults with type 2 diabetes.^21^ In these studies, HbA1c was found to fluctuate seasonally with highest values recorded in winter. Insulin resistance, as calculated using the HOMA-IR equation, has been shown to be greater in winter compared to summer in pregnant women both with and without GDM.^3,22^ The cause of the variation in insulin resistance is not clear, although vitamin D has been proposed as a potential explanation. Low vitamin D levels show seasonal variation and are associated with development of type 2 diabetes.^23^ Vitamin D deficiency has been shown to significantly increase the risk of developing GDM^24^ and a meta-analysis of trials of vitamin D supplementation in women with GDM showed that supplementation improved glycaemic control, decreasing both fasting plasma glucose and HbA1c.^25^ However, a recent study analysing the impact of both season and vitamin D on glucose homeostasis showed that they independently impact glucose homeostasis suggesting that although vitamin D does influence seasonal variation in insulin resistance, it is not the sole contributor.^22^ Further research would be required to identify other contributing factors.

### 4.3 | Diet and exercise

Before pharmacological management, all women are advised to adopt a low glycaemic index diet and engage in physical activity but the ability to engage in these treatments may vary seasonally. Nutrient intake varies in pregnancy according to season,^26^ and differences in dietary intake throughout the year including around holidays such as Christmas and Easter have been shown to contribute to seasonal variation in glycaemic control in type 2 diabetics.^27^ Physical activity levels vary throughout the year with peak activity in summer^28^ and a qualitative study assessing influences on physical activity in pregnancy identified unfavourable weather as a barrier.^29^ Support for indoor exercise could reduce the impact of the weather and a trial home-based indoor exercise has shown it to be beneficial in terms of improved glycaemic control in women with GDM.^30^

Our study has several important strengths including a large cohort managed at a single centre according to standardised protocols. The dataset was generated from hand-searching of a detailed electronic records system from documentation recorded at the time of clinical encounters. We use non-parametric dynamic additive models as a flexible way to determine the risks of outcomes relative to baseline risk at any time-point in the year while avoiding making any a priori assumptions about the relationship of risk to time or introducing arbitrary time divisions within the annual cycle.

There are limitations of this study. Some data were incomplete, most notably maternal BMI was not recorded for 23.3% of the participants. We excluded women who delivered preterm infants from this analysis (typically 9.9% of our population) as the risk of neonatal hypoglycaemia is much higher after preterm delivery. However, it is possible that this may have excluded women with most severe hyperglycaemia or coexisting related conditions such as preeclampsia leading to an underestimation of the risk for this population. Although the size of the cohort was large overall, subset analyses included a more limited number of women. The variables collected do not enable calculation of maternal insulin resistance. Further research is needed to determine whether these findings are replicated in other settings and whether adjustments such as vitamin D supplementation or supporting indoor exercise could improve glycaemic control and reduce pharmacological treatment requirements in women diagnosed with GDM in winter.

The temporal variation in treatment requirements and neonatal outcomes in GDM observed here is likely multifactorial. It is possible that the observed variance is primarily attributable to maternal behavioural differences throughout the year, particularly in diet/exercise patterns. However, it is also possible that GDM is less comprehensively monitored and treated in summer months, potentially due to service or lifestyle-related factors. This possibility appears less likely as previous studies indicate no increase in average birthweight during times of year when insulin treatment is reduced.^5^ Future studies that include results of continuous glucose monitoring in GDM may help to further assess detailed correlations between glycaemic control and insulin prescription.

In summary, this study demonstrates that the requirement for insulin treatment in GDM varies significantly throughout the year, even in a relatively temperate climate. We also identified temporal variation in risk of neonatal hypoglycaemia, particularly involving infants of mothers with correspondingly high treatment requirements. Improved understanding of temporal effects allows better access to pharmacological treatment and optimal surveillance to prevent neonatal complications.

## Supplementary Material

Supp 1

Supp 2

## Figures and Tables

**TABLE 1:** Maternal demographic data, obstetric history and management (*n* = 791).

Patient demographics	Mean	±SD
Maternal age (years)		
	33.6	±5.3
	*Frequency*	*Percentage*
Ethnicity		
Caucasian	574	72.6
Asian	156	19.7
Black	19	2.4
Other	38	4.8
Unknown	4	0.5
Smoking		
Yes	58	7.3
No	688	87.0
Unknown	45	5.7
Maternal BMI		
<25	202	25.5
25–29	162	20.5
30–34	117	14.8
35–39	77	9.7
≥40	49	6.2
Unknown	184	23.3
Obstetric history	Frequency	Percentage
Parity		
0	356	45.0
1	271	34.3
≥2	164	20.7
Previous GDM		
Yes	112	14.2
No	323	40.8
Not applicable	356	45.0
Previous LSCS		
Yes	139	17.6
No	296	37.4
Not applicable	356	45.0
Previous vaginal delivery		
Yes	317	40.1
No	118	14.9
Not applicable	356	45.0
GDM diagnosis	Frequency	Percentage
Timing of OGTT		
<24 weeks	79	10.0
24–28 weeks	333	42.1
>28 weeks	365	46.1
No OGTT	14	1.8
GDM diagnosis	Frequency	Percentage
	*Mean*	±*SD*
OGTT results		
0 min	4.8	±0.7
60 min	10.6	±1.5
120 min	7.6	±1.6
HbA_1c_ at diagnosis (%)	5.4	±0.6
HbA_1c_ at diagnosis (mmol/ mol)	35.8	±4.9
Treatment	Frequency	Percentage
Type of treatment		
Diet	391	49.4
Metformin only	93	11.8
Insulin only	195	24.7
Both	112	14.2
Type of insulin		
Short-acting	171	
Long-acting	268	
Both	135	
Treatment-doses required	Median	IQR
Mean total daily metformin dose at 36 weeks (g; *n* = 205)	1.5	1.0–2.0
Median insulin dose at 36 weeks (units/day; *n* = 307)		
Short-acting	6.0	1.0–19.0
Long-acting	8.0	4.0–16.0
Delivery	Frequency	Percentage
Delivery mode		
Spontaneous vaginal delivery	368	46.5
Instrumented vaginal delivery	104	13.1
Elective caesarean section	184	23.3
Emergency caesarean section	135	17.1
Estimated blood loss (ml)	Mean 503	±SD ±384.9

**TABLE 2:** Neonatal outcome data (*n* = 790).

Neonatal outcomes	Frequency	Percentage
Sex		
Female	363	45.9
Male	427	54.1
Neonatal hypoglycaemia		
Yes	360	45.6
No	424	53.7
Not recored	6	0.8
Admission to NICU		
Yes	99	12.5
No	691	87.5
Neonatal jaundice		
Yes	129	16.3
No	658	83.3
Not recorded	3	0.4
	*Mean*	±*SD*
Birthweight (g)	3308	±425
Lowest blood glucose (mmol/L)	2.6	±0.6

## What is already known?

- Rates of GDM and related pregnancy outcomes, including birthweight centile and emergency caesarean section rates, show seasonal variation
- It is unclear if temporal variation also influences maternal treatment requirements or other neonatal complications

## What this study has found?

- Women diagnosed in November were more likely to need insulin treatment. In winter, women required 19 units/day more insulin compared to summer-diagnosed women
- The incidence of neonatal hypoglycaemia also varied with season

## What are the clinical implications of the study?

- Insulin treatment requirements in GDM and neonatal hypoglycaemia rates show significant annual variation, even in a temperate climate.
