# Supplementary material for: Temporal variations in maternal treatment requirements and early neonatal outcomes in patients with gestational diabetes
Source: Diabet Med. Author manuscript; Available in PMC 2024 Jan 11. (PMC10782837; doi:10.1111/dme.14596)
Supplement: Supp 1 [file NIHMS1907922-supplement-Supp_1.docx]

**Supplementary material**

Supplementary Figure 1: The impact of temporal variation upon requirement for insulin treatment using generalised additive modelling (GAM)

A) X axis commencing 1st January p(overall trend)<0.05

B) X axis commencing 1st July p(overall trend)<0.05
