# Supplementary figures and images for: Temporal variations in maternal treatment requirements and early neonatal outcomes in patients with gestational diabetes
Source: Diabet Med. Author manuscript; Available in PMC 2024 Jan 11. (PMC10782837; doi:10.1111/dme.14596)

## Slide 1
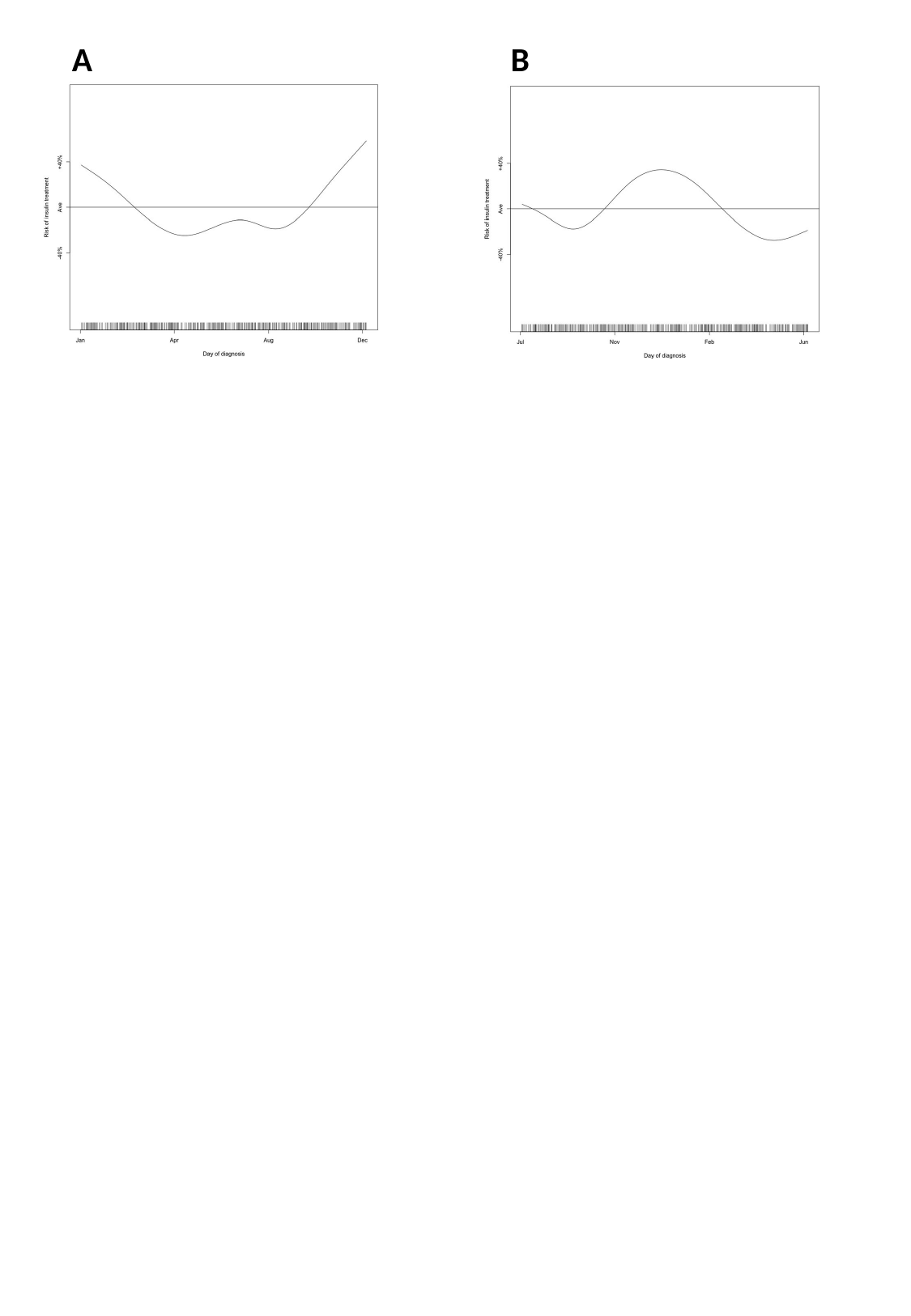

A
B

Supplement: Supp 2 [file NIHMS1907922-supplement-Supp_2.pptx]
